# Performance of pelican optimizer for energy losses minimization via optimal photovoltaic systems in distribution feeders

**DOI:** 10.1371/journal.pone.0319298

**Published:** 2025-03-12

**Authors:** Zuhair Alaas, Ghareeb Moustafa, Hany Mansour

**Affiliations:** 1 Department of Electrical and Electronics Engineering, Faculty of Engineering and Computer Science, Jazan University, Jizan 45142, Saudi Arabia; 2 Department of Electrical Engineering, Faculty of Engineering, Suez Canal University, Ismailia, Egypt; Graphic Era Deemed to be University, INDIA

## Abstract

In distribution grids, excessive energy losses not only increase operational costs but also contribute to a larger environmental footprint due to inefficient resource utilization. Ensuring optimal placement of photovoltaic (PV) energy systems is crucial for achieving maximum efficiency and reliability in power distribution networks. This research introduces the Pelican Optimizer (PO) algorithm to optimally integrate solar PV systems to radial electrical distribution grids. The PO is a novel bio-inspired optimization algorithm that draws inspiration from pelicans’ intelligence and behavior which incorporates unique methods for exploration and exploitation, improving its effectiveness in various optimization challenges. It introduces a hyper-heuristic for phase change, allowing the algorithm to dynamically adjust its strategy based on the problem’s characteristics. The suggested PO aims to reduce the energy losses to the possible minimum value. The developed PO version is tested on the Ajinde 62-bus network, a practical Nigerian distribution system, and a typical IEEE grid with 69 nodes. The simulation findings demonstrate the enhanced PO version’s efficacy, showing a significant decrease in losses of energy. With the Ajinde 62-node grid, the suggested PO version obtains a substantial 30.81% decrease in the total energy loss expenses in contrast to the initial scenario. Similarly, the IEEE 69-node grid achieves a significant decrease of 34.96%. Additionally, the model’s findings indicate that the proposed PO version performs comparably to the Differential Evolution (DE), Particle Swarm Optimization (PSO), and Satin bowerbird optimizer (SBO) algorithms.

## Introduction

Recently, using renewable energy resources (RERs) has become more consequential as an approach to the problems caused by rising temperatures, climate change, a blockchain-based microgrid [1], network restructure upgrading [2], as well as the depletion of conventional energy sources [3]. Based on the International Renewable Energy Agency’s forecasts, RERs are expected to produce approximately 85% of all energy generated by 2050 [4]. Solar PV systems are the second most widely used RERs globally, after wind power sources [5]. The Earth receives an average solar radiation level of 1367 W/m2, with a total global absorption of solar energy estimated at around 1.8 x 1011 MW. That abundant as well as inexhaustible resource of energy surpasses the global demand for electricity by a significant margin [6]. Solar energy is anticipated to constitute around 20% of global energy generation by 2050 [4]. Presently, substantial investments have been directed towards renewable energy, with a focus on solar power. An example of this is the Benban project in Egypt with size of 1.8 GW [7].

### Motivation

In distribution grids, excessive energy losses increase operational costs and contribute to a larger environmental footprint due to inefficient resource utilization. Typically, solar PV units are installed on distribution grids to deliver electricity alongside the main utility grid, which can expose the system to a variety of technical issues [8]. However, the accurate forecasting tools are highly required for the efficiency analysis of their output energies via Artificial intelligence (AI)-based systems [9]. Both irregular energy and unpredictable behavior of such decentralized solar panels present challenges in evaluating and optimizing their technical advantages for distribution networks [10]. A high penetration of PV systems could either yield significant advantages or lead to serious adverse effects with the functioning of electricity distribution grids [11]. Them main advantages for solar energy systems installation involve lowering energy production expenses, enhancing grid dependability, mitigating harmful carbon emissions, and easing the strain on transmission system capacity [12]. However, with irregularity of solar power generating profiles can disrupt that normal functioning for distribution networks, resulting various operational issues, notably voltage variations, increased real energy losses, as well as excessive reactive energy losses [13]. The PV cells/modules have been usually represented via their equivalent circuits including the single-diode [14], double-diodes [15], and triple diode models [16]. Nevertheless, ensuring the optimum allocation for solar PV systems is essential for meeting its intended goals during the installation of RERs on distribution systems. Optimal allocation of PV units entails identifying both the appropriate positions as well as the rating for connected PV units.

## Literature survey

Researchers have considerably worked on and applied metaheuristic optimization techniques to tackle the issues related to optimal installation of these systems in distribution grids. A comprehensive review of optimal placement PV units in power grids has been presented in [17]. In [18], the researchers have explored the use of PV units and wind power plants as RERs in distribution grids. They have employed the white shark optimization (WSO) approach to determine the optimal sites and sizes of these systems by balancing exploration as well as exploitation towards global optimization purposes. The analysis has been conducted on IEEE 33-node, 69-node, and 85-node distribution networks to assess the effectiveness of the suggested method. The results showed that WSO significantly lowered the real power loss, enhanced voltage characteristics, as well as surpassed alternative approaches. The study demonstrated that raising the number of installed PV systems led to considerable decrease in losses reached to 52.267%. Indeed, raising the number of installed wind systems achieved a 93.2% reduction in power losses. Furthermore, the integration of solar power and wind turbine systems resulted in noticeable improvements in voltage profiles.

In [19], a two- methodologies have been proposed to determine the optimum locations and capacities for PV units and distribution static synchronous compensators (DSTATCOM). For the first approach, the voltage loss sensitivity index has been applied for determining the important priority locations for installation of the solar units and DSTATCOM. For the second approach, a hybrid technique involving the lightning attachment procedure optimizer and the equilibrium optimization algorithm, have been suggested to find the optimal buses as well as capacities of installed units. The seasonal changes in irradiance and load demand have been considered. The suggested methods have been executed on a 118-bus distribution network.

In addition, the authors in [20] have investigated the optimum capacity as well as placement of solar energy units in a modified IEEE 14-node grid. They employed a hybrid approach involving the genetic algorithm (GA) and the particle swarm optimizer (PSO). The fitness function has been designed to reduce the whole power loss, boost voltage and frequency values. Their study incorporated two cases to evaluate the impact of integrating PV systems, changes in load consumption, and environmental factors on optimizing system size. In [21], a novel algorithm called artificial ecosystem-based optimization-opposition-based learning (AEO-OBL) has been suggested. The main objective of AEO-OBL was to find the optimum placement and ratings of the installed RERs in distribution networks to reduce whole active losses. The unstable nature of RERs has been considered. The proposed AEO-OBL has been executed to the IEEE 33-node and 85-node networks. Indeed, a two-stage optimization approach has been suggested to address the problem of solar PV allocation [22]. A mixed-integer quadratic approximation has been applied to determine the optimum places for PV systems then an interior point technique has been implemented to obtain the optimum ratings of these systems. The proposed approach has been tested on two different distribution networks: IEEE 33, 85-bus.

In [23], a multi-objective hybrid approach included teaching–learning-based optimization technique and the grey wolf optimization technique has been proposed to address the optimum placement and sizing of RERs in distribution grids. The proposed approach has been utilized to determine the optimal sites and capacities of solar and wind systems. The fitness function has been formulated to minimize the active losses and enhance the grid reliability. The proposed technique has been implemented to IEEE 33-node and 69-bus grids. In [24], the Gorilla Troop’s Optimization (GTO) technique has been used for solving the optimum placement issue of solar units integrated with DSTATCOM on a 94-node grid. Three objectives have been considered within a multi-objective framework, encompassing the overall yearly expenses, voltage variations, and grid stability. In [25], the Artificial Rabbits Optimization Algorithm (AROA) has been proposed to allocate the PV/STATCOM resources at different times throughout the day. The objective function has been formulated to minify both the energy losses and grid voltage variations across various 24-hour load scenarios. The presented AROA has been applied to a standard IEEE 33-node distribution network.

## Gaps in literature

Despite significant progress, gaps remain in the existing methodologies:

1. Limited adaptability to dynamic operational conditions, such as hourly load variations and irradiance changes.
2. Inadequate exploration-exploitation balance in optimization algorithms, leading to suboptimal convergence rates.
3. Scarce comparative evaluations of emerging optimization methods with established algorithms.
4. Insufficient application of bio-inspired optimization techniques for addressing the specific challenges of solar PV placement.

### Novelty

This study addresses the identified gaps by introducing the Pelican Optimizer (PO), a novel bio-inspired algorithm modeled on pelican hunting behavior. The PO incorporates hyper-heuristic mechanisms for dynamic adaptability, enhancing both exploration and exploitation phases of the optimization process. This algorithm aims to minimize energy losses by optimizing the placement and sizing of solar PV systems in radial power distribution grids. It also seeks to validate its effectiveness through comprehensive testing on two distribution networks: the practical Ajinde 62-node Nigerian grid and the IEEE 69-node grid. Moreover, the study compares the PO performance with well-established algorithms such as DE, PSO, and SBO from the state of the art. The proposed PO algorithm demonstrates significant reductions in energy losses and improved network performance metrics, providing a robust and efficient solution for PV system integration.

### Why PO is used in the proposed work

PSO is a widely adopted algorithm due to its simplicity and ability to handle a variety of optimization problems. However, it suffers from several limitations as it often converges prematurely, especially in multi-modal or highly constrained optimization problems [26], due to the loss of diversity in the swarm. It uses static parameters for velocity and position updates, which limits its ability to adapt dynamically to the problem’s characteristics [27]. Also, DE is known for its robustness and efficiency in solving continuous optimization problems, but it is highly dependent on parameter tuning (e.g., mutation and crossover rates). DE can become trapped in local minima when dealing with multi-modal functions with numerous peaks and valleys [28]. Moreover, SBO is a bio-inspired algorithm that mimics the mating and habitat construction behaviors of bowerbirds. While promising, has a strong focus on exploration during the initial stages, but its exploitation capabilities in later stages are limited. SBO struggles to maintain performance consistency when applied to large-scale problems with numerous decision variables.

The PO algorithm was developed by P. jovský and M. Dehghani in [29]. The pelican is a large bird with a lengthy beak and a big pouch in its sore, which it uses to constipate and eat victims. These birds live in groups of several hundred and are social animals. Pelicans typically weigh in the range from 2.75 kg to 15 kg, stand around 1.06 to 1.83 m height, and have a wingspread ranging from 0.5 to 3 m. They mainly eat fish but consume frogs, turtles, crustaceans, and sometimes shellfish when hungry. Pelicans often work together to hunt. They dive from 10–20 meters when they find their prey, sometimes even lower. They spread their wings over the plane of the water to push the fish into shallower areas, making it easier to capture them. While catching fish, much water fills the pelican’s beak, so they must tip their head forward to drain the water before swallowing it. Pelicans’ hunting behavior and technique are complex processes that have made them skilled hunters. The main reason for creating the proposed PO is to model this strategy.

While existing techniques like PSO, DE, and SBO have their merits, their limitations in handling dynamic, complex, and large-scale optimization problems. The PO algorithm addresses these limitations by introducing innovative mechanisms inspired by pelican hunting behavior. The PO method efficiently identifies the most relevant solutions within the search domain and quickly converges with optimal solutions. The algorithm is easy to apply. The efficacy of the PO’s has been verified through various scenarios, including multiple single modal and multimodal functions [29]. These verifications highlight the algorithm’s capability to handle issues related to global optimization. These characteristics make it suitable for various practical applications, such as demand response approach for microgrid energy management [30], PV array dynamic reconﬁguration [31], and cluster head choice in wireless systems [32].

## Paper contributions

- This study presents a novel optimization algorithm called Pelican Optimizer (PO), specifically designed for addressing the issue of optimum placement and sizing of solar PV systems in power distribution networks. The principal contributions of this article are outlined below:
- The paper proposes the (PO), a novel bio-inspired algorithm modeled on pelican hunting behavior, for optimizing the location and capacity of solar PV systems in power distribution grids.
- The primary objective of the problem is to minimize energy losses in distribution feeders by finding the optimal sites and sizes of connected solar PV systems using the PO.
- The designed PO method attained substantial decreases in energy losses in the Ajinde 62-node grid and the IEEE 69-node grid, outperforming other techniques like Differential Evolution (DE), PSO, and the Satin Bowerbird Optimizer (SBO).
- The proposed PO algorithm was validated on two distinct electrical distribution systems: a practical Nigerian grid (Ajinde 62-node grid) and the standard IEEE 69-node grid. The algorithm showed impressive results, achieving a 30.81% and 34.96% reduction in energy losses, respectively.
- The study demonstrated that the PO outperformed other well-known algorithms (DE, PSO, SBO) not only in reducing energy losses but also in terms of convergence speed, efficiency, and stability, confirming its potential as an effective tool for optimization in power grids.

## Problem formulation: Solar PV systems allocation in distribution systems

Integrating solar PV systems into distribution grids requires determining the optimal allocation of sites and capacity. These variables substantially impact the determination of power losses and energy losses related to distribution grids.

### Objective model

In distribution grids, it is essential to consider power losses and energy dissipation as heat during the transmission and distribution of electrical power. These energy losses can reduce the system’s efficiency, leading to higher expenses and a more significant environmental impact. Equation (1) quantifies how optimizing the solar PV system model in distribution grids can help reduce energy losses and improve the system’s overall energy efficiency as follows [12]:


$$DL_{Energy}=\sum_{\text{h}=\text{1}}^{{\text{N}}_{\text{h}}}{\text{HL}}_{h}$$ (1)

where *DL*_*Energy*_ expresses the daily energy losses. h refers to hour while *N*_*h*_ represents the total number of hours, and *HL*_*h*_ signifies power losses lasting one hour (*h*). The following may be formulated to illustrate it as follows [12]:


$$\text{HL}={\left(\sum_{\text{B}=\text{1}}^{{\text{N}}_{\text{B}}}{\left({\text{I}}_{B}\right)}^{2}\times R_{B}\right)}_{h}$$ (2)

where the whole network branch’s number is denoted by *N*_*B*_ and the current flowing through each branch is represented by *I*_*B*_ while *R*_*B*_ refers to the branch resistance.

### Constraints of the solar PV systems

The optimization model being examined includes two categories of design variables: the exact positions and capacities of the solar PV systems. When it comes to positioning solar PV systems, they have the flexibility to be connected to any bus within the system. The following mathematical equation describes this flexibility [33]:


$${\left(2\leq Pos_{PV,J}\leq N_{bus}\right)}_{J=1:N_{PV}}$$ (3)

*Pos*_*PV*_ refers to the candidate positions to which solar PV systems can be installed. All buses except the slack bus are candidate locations for solar PV connection. *N*_*PV*_ represents the greatest number of solar PV units that can be considered.

Additionally, there is a maximum limit restriction that applies to each potential solar PV system as follows [33]:


$${\left(0\leq Size_{PV,J}\leq Size_{PV,\max}\right)}_{J=1:N_{PV}}$$ (4)

where *Size*_*PV,J*_ signifies the capacity of the solar PV unit connected at the selected bus (*Pos*_*PV,J*_), whereas *Size*_*PV,max*_ indicates the maximum permissible capacity.

### Constraints due to the electrical distribution system

Furthermore, it is essential to guarantee that the voltages are kept within the acceptable thresholds at each bus. The following constraint may serve as a representation of this need in mathematical terms [34]:


$${\left(V_{n,\min}\leq V_{n}\leq V_{n,\max}\right)}_{n=1:N_{bus}}$$ (5)

The parameters *V*_*n,min*_ and *V*_*n,max*_ express the lowest and greatest voltage thresholds at the buses within an allowable limit of 5%. Maintaining voltage values within this acceptable limit is crucial to prevent voltage instability or apparatus damage [35]. Indeed, complying with security limitations when transmitting electricity across the system is crucial. One way to achieve this is by constraining the current flow to acceptable ranges across each network branch. This can be represented as follows [36]:


$${\left(I_{B}\leq I_{B,\max}\right)}_{B=1:N_{B}}$$ (6)

where, *I*_*B,max*_ and *I*_*B*_ express the maximum thermal capability and the line current while *N*_*B*_ refers to the total grid branches number. By implementing these restrictions, the network guarantees that the flow of electric current remains within acceptable operational conditions, preventing excessive loads and potential equipment malfunctions. Furthermore, it is essential to sustain the equilibrium between real and reactive power during every operational hour (*h*), which can be expressed using the following conceptual framework system [33]:


$$\sum_{i=1}^{N_{bus}}Pd_{i,h}+HL_{h}=P_{Grid,h}+{\left(\sum_{J=1}^{N_{PV}}P_{PV_{PosPV,J}}\right)}_{h}$$ (7)

$$\sum_{i=1}^{N_{bus}}Qd_{i,h}+QL_{h}=Q_{Grid,h}$$ (8)

In this framework, *P*_*di*_ and *Q*_*di*_ are the real and reactive electricity load at each node (*i*), respectively. The terms *HL* and *QL* refer to the active and reactive losses. *P*_*Grid*_ and *Q*_*Grid*_ represent the whole active and reactive power delivered from the main, respectively. By incorporating these constraints on power flow balance, the grid can sustain stability and ensure the proper balance of real and reactive power transmitted and used. It is essential to ensure efficient and reliable operation to minimize losses and optimize the overall performance of the distribution grid [37].

## PO for enhancing distribution feeders with optimal PV systems

The pelican hunt’s behavior and tactics served as the inspiration for the PO, a novel metaheuristic technique [29]. The PO approach involves two steps to find the global maximum of the fitness function by emulating the behavioral patterns of a pelican. First, it involves moving towards the prey (exploration stage), and then it involves gliding over the water plane (exploitation stage).

### Exploration stage

In the initial stage, groups of pelicans locate their prey and then go towards it. The space between the pelicans and the victim affects their fitness, which in turn updates their position. By repeating and moving, the pelicans can reach and gather at the highest possible position. Equation (9) mathematically formulates the moving tactics of the pelican towards its prey [29].


$$X_{m,n}^{P1}= \{\begin{array}{cc} x_{m,n}+rand\times (p_{n}-r\times x_{m,n}) & F_{p}<F_{m}\\ x_{m,n}+rand\times (x_{m,n}-p_{n}) & else \end{array}$$ (9)

where $x_{m,n}$ represents the updated position of the *m*^th^ pelican in the nth dimension based on the 1st stage. “$p_{n}$” refers to the victim’s position in the nth dimension, while *Fp* denotes the value of its fitness function. The parameter “*r*” expresses a random value that can be either 1 or 2. It is selected arbitrarily for each iteration and each fellow. When the magnitude of “*r*” is set to 2, it leads to greater movement for a pelican. This permits the member to explore new areas within the search space. As a result, parameter r directly influences the scanning ability of the search space in PO exploration.

The proposed PO determines whether to accept a new location for a pelican based on the improvement in the value of the fitness function at that location. This updating method, known as efficient updating, prevents the algorithm from moving to suboptimal regions. Equation (10) describes this process as follows [29]:


$$X_{m}= \{\begin{array}{cc} X_{m,n}^{P1} & F_{m}^{P1}<F_{m}\\ X_{m} & else \end{array}$$ (10)

where *X*_*m*_ represents the updated state of the *m*^th^ pelican and $F_{m}^{P_{1}}$ is its fitness function evaluation based on stage 1.

### Exploitation stage

Upon arriving the water’s plane, pelicans extend their wings to propel the fish and subsequently capture the prey in their neck pouch. This method leads to a higher capture rate of fish in the targeted region. The suggested PO algorithm successfully converges to optimal locations within the hunting region by modelling pelicans’ behavior. This procedure boosts the local search ability and the exploitability of PO-based systems. In simple terms, the algorithm needs to analyze the positions nearby the pelican’s position to reach a more optimal result. Equation (11) represents the quantitative simulation of pelicans’ hunting behavior [29].


$$X_{m,n}^{P2}=x_{m,n}+k\times (1-\frac{t}{T})\times (2\times rand-1)\times x_{m,n}$$ (11)

where $X_{m,n}$ represents the updated state of the m^th^ pelican in the nth dimension based on the second stage. *k* is a fixed magnitude that is precisely 0.2. “*t*” signifies the current iteration, and*T* refers to the final iteration. The coefficient $"k\left(1-\frac{t}{T}\right)"$ represents the region radius of $X_{m,n}$.This coefficient significantly enhances the exploitability of PO, bringing it nearer to the global optimum result. During the preliminary iterations, the factor has a substantial value, leading to the consideration of a larger domain around each member.

After each iteration, the approach renews the pelican’s location and determines the optimum choice. Upon reaching the maximum iteration limit or satisfying the convergence requirement, the approach stops and provides the optimization results.

**Fig 1:**
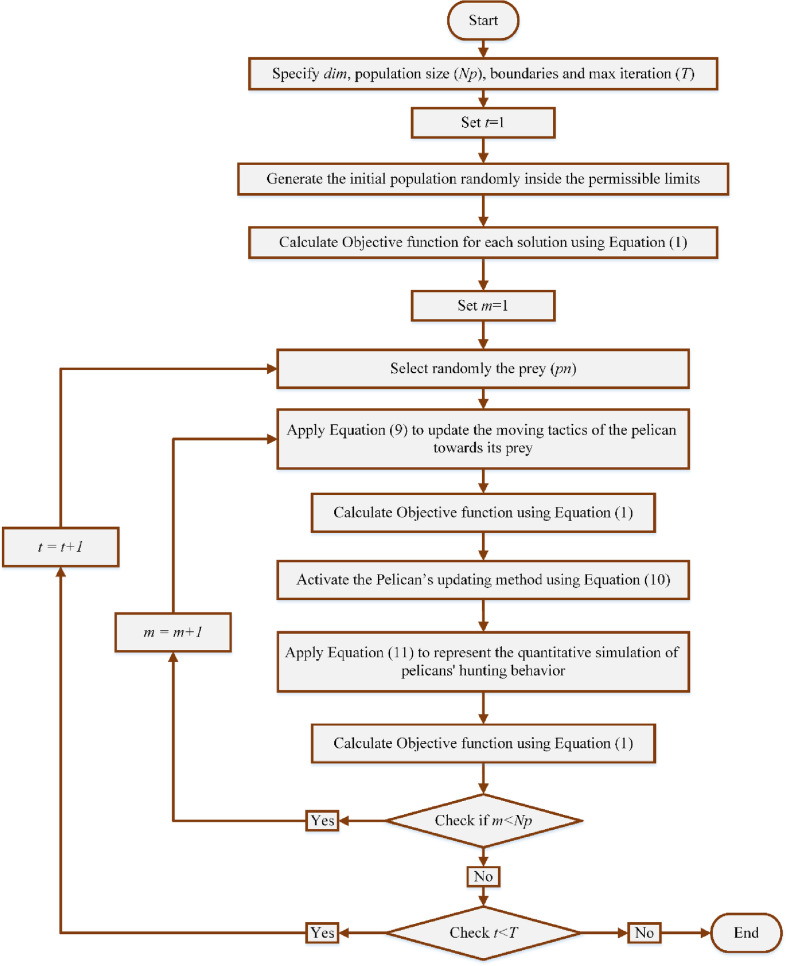
PO Flowchart for photovoltaic integrations in distribution feeders.

Fig 1 illustrates the steps of the proposed enhanced PO version, as detailed in the previously stated stages. Also, Algorithm 1 displays pseudo-code of the designed PO algorithm to minimize energy losses in distribution networks by optimizing the placement and sizing of solar PV systems.

**Table:** 

**Algorithm 1: PO Pseudo-Code**
**Problem:** Minimize energy losses in distribution networks by optimizing the placement and sizing of solar PV systems.
**Inputs:**
• Problem-specific parameters: Bus and branch data of the system under study; number of buses (*N*_*bus*_); number of branches (*N*_*B*_); maximum PV capacity (*Size*_*PV,max*_); voltage limits (*V*_*n,min*_ and *V*_*n,max*_). • PO parameters: population size (*N*_*P*_) and maximum iterations (*T*).
**Outputs:**
• Optimal positions of PV systems (*Pos*_*PV*_), Optimal sizes of PV systems (*Size*_*PV*_), and corresponding minimum energy losses.
**Step 1: Initialize Parameters**
1. Insert bus and branch data of the system under study. 2. Set population size size (*N*_*P*_) and maximum iterations (*T*). 3. Initialize *N*_*P*_ pelicans randomly in the solution space. 4. Assign each pelican’s position represents candidate positions and capacities for PV systems. 5. Ensure all solutions satisfy problem constraints regarding the design variables (e.g., Equations 3 and 4). 6. Run the 24-hour load flow after installing the positions and capacities for PV systems considering their PV hourly variations. 7. Evaluate the fitness of each pelican using the energy losses defined in Equations (1) and (2). 8. Ensure the hourly operational problem constraints (Equations 5–8) and if there is exceed, set the fitness of this pelican at a high value. 9. Identify the best solution (*X*_*best*_) in the initial population.
**Step 2: Optimization Process**
10. For t = 1 to T: 11. Apply the exploration phase to explore the search space by moving towards the prey using Equation (9). 12. Assign each pelican’s position represents candidate positions and capacities for PV systems. 13. Ensure all solutions satisfy problem constraints regarding the design variables (e.g., Equations 3 and 4). 14. Run the 24-hour load flow after installing the positions and capacities for PV systems considering their PV hourly variations. 15. Evaluate the fitness of each pelican using the energy losses defined in Equations (1) and (2). 16. Ensure the hourly operational problem constraints (Equations 5–8) and if there is exceed, set the fitness of this pelican at a high value. 17. Apply the updating method to prevent moving to suboptimal regions using Equation (10) 18. Apply the exploitation phase to refine the search around promising areas using Equation (11) 19. Assign each pelican’s position represents candidate positions and capacities for PV systems. 20. Ensure all solutions satisfy problem constraints regarding the design variables (e.g., Equations 3 and 4). 21. Run the 24-hour load flow after installing the positions and capacities for PV systems considering their PV hourly variations. 22. Evaluate the fitness of each pelican using the energy losses defined in Equations (1) and (2). 23. Ensure the hourly operational problem constraints (Equations 5–8) and if there is exceed, set the fitness of this pelican at a high value. 24. If a pelican’s new position has a better fitness than *X*_*best*_, upgrade the best solution (*X*_*best*_) in the population. 25. End For
Step 3: Output Results
26. Return the best solution (*X*_*best*_), which includes the optimal positions systems (*Pos*_*PV*_), Optimal sizes of PV systems (*Size*_*PV*_), and corresponding minimum energy losses.

## Outcomes and discussion

**Fig 2:**
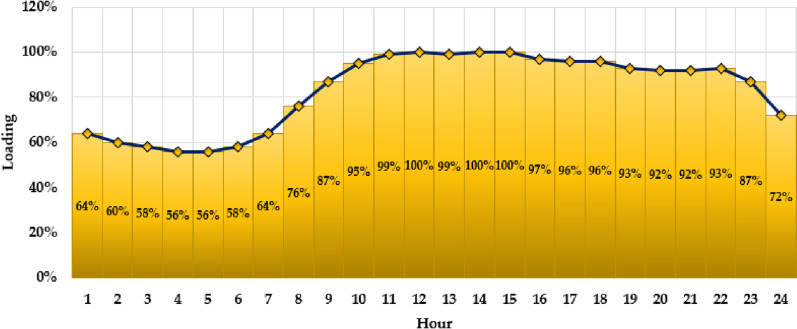
Hourly loading variations.

In this study, the suggested PO approach is executed on two distribution grids: the Ajinde 62-node system [38], a practical Nigerian distribution grid, and the IEEE-69 node grid [39]. The maximum capacity for integrating solar PV system in both grids is specified at 2000 kW, with the greatest allowance of three connections. Furthermore, the hourly demand fluctuations, represented as a proportion of the maximum demand situation, are considered, as shown in Fig 2 [33].

The goal of this paper is to improve the integration of solar PV units in distribution grids by minimizing the total losses, as defined in Equation (1). IEEE 1547 recommends that each solar PV system operates at a unity power factor [40]. Also, the whole size to be connected to the grid is constrained to 60% of the peak demand [41].

Table 1 shows the maximum solar PV capacity, and voltage limits for connecting the solar PV system to power grids. The applications of the PO involve 100 iterations, each repeated 20 times with 30 solution agents.

**Table 1:** PV allowable capacity and voltage boundaries.

Parameter	Value
Maximum acceptable capacity *(Size*_*PV,max*_)	2000 kW
Minimum voltage constraint at each bus $\left(V_{n,min}\right)$	0.95 p.u.
Minimum voltage constraint at each bus $\left(V_{n,max}\right)$	1.05 p.u.

### First grid: Ajinde 62-node

**Fig 3:**
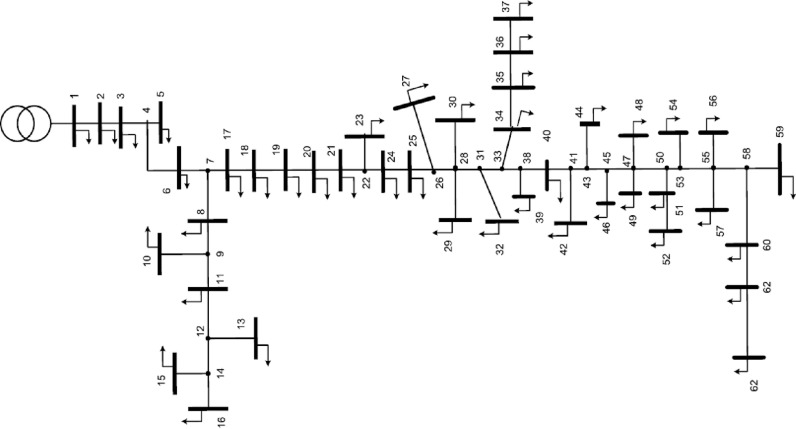
Single-Line scheme of Ajinde 62-node grid.

The system being assessed consists of 62 nodes and 61 branches. The data for this grid was taken from the Ibadan Electricity Distribution Company of Nigeria (IBEDC) [38]. The total real and reactive loads on the grid were measured to be 2.07 MW and 1.29 MVAr, respectively. The voltage maintained for the network was 11 kV. Fig 3 shows the one-line scheme of the grid.

**Fig 4:**
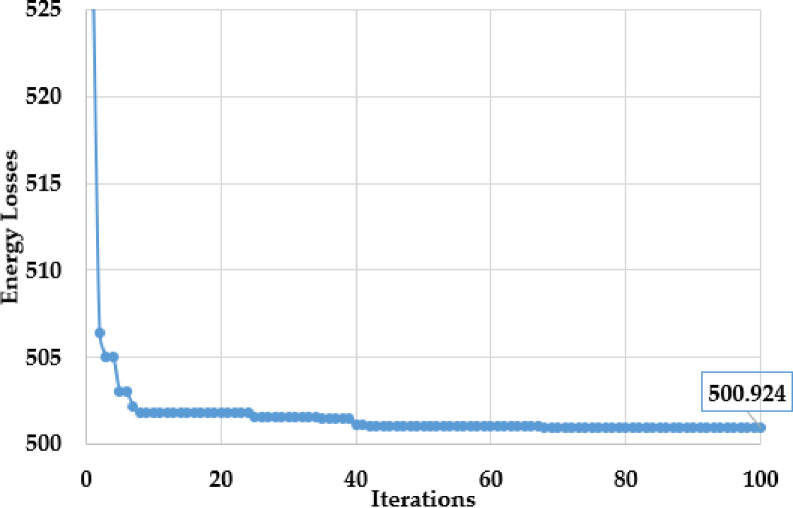
Convergences of PO algorithm for the Ajinde 62-node Nigerian grid.

To minify the energy losses, the PO method is performed to integrate solar PV systems in the first assessed grid. The outcomes of optimal placement and sizing of solar PV systems in the first grid are detailed in Table 2. The convergence features of the proposed PO method for addressing this problem are shown in Fig 4. The optimal allocation is selected at nodes 33, 53, and 60 with matching capacities of 679 kW, 426 kW, and 276 kW, respectively. The enhanced PO approach decreases energy losses from 723.988 MWh to 500.9237 MWh with a significant decrease of 30.81%.

**Table 2:** Achieved outcomes of solar PV units’ allocation by PO for the Nigerian system.

Parameters	Initial	Placement (bus)	Capacity (kW)
Solar PV units’ integration	–	33	679
	–	53	426
	–	60	276
Total photovoltaic installed Capacity	–	1381 kW	
Energy losses (MWh)	723.988	500.9237	

**Fig 5:**
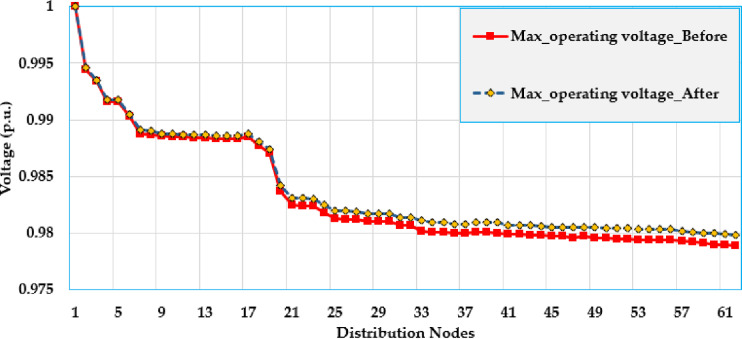
Maximum voltage values over the grid using PO algorithm of the Ajinde 62-bus Nigerian system.

**Fig 6:**
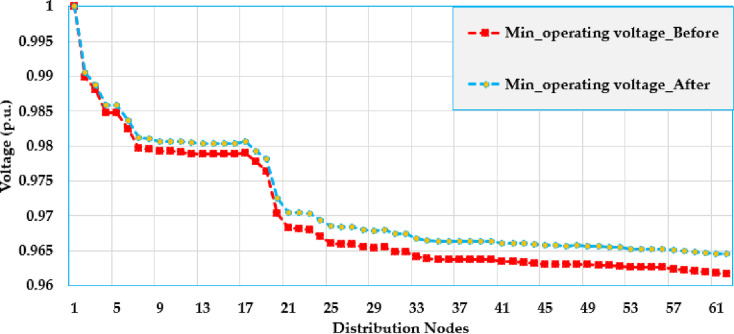
Minimum voltage values over the grid using PO algorithm of the Ajinde 62-bus Nigerian system.

Figs 5 and 6 show the voltage profile at different nodes in the test grid, illustrating how the voltage is distributed across the system. The figures display the lowest and highest voltage magnitudes noted at each bus. As shown, the integration of solar PV system has a significant impact on the voltage levels and leads to considerable improvements compared to the baseline.

To ensure the efficacy of the proposed PO method, a comparative analysis utilizing alternative documented methodologies, DE, PSO, and SBO, is tabulated in Table 3. The behavior of the suggested PO in terms of lowest, average, highest, and standard deviation of the objective function minimization confirms the validity, stability, and effectiveness of the suggested PO approach.

**Table 3:** Comparisons between the PO algorithm with reported techniques for the Nigerian system.

Algorithms	Energy losses (MWh)				
	Minimum	Average	Maximum	Standard Deviation	Percentage Improvement based on the average losses
DE [33]	500.9412	501.2314	501.5581	0.1667	0.0039%
PSO [33]	501.7297	503.0422	505.0854	0.8191	0.3638%
SBO [33]	500.9873	501.4749	502.2436	0.3303	0.0524%
PO Algorithm	500.9237	501.2119	501.9493	0.3240	

As displayed in that table, PO achieves the lowest energy losses at 500.9237 MWh, slightly better than DE (500.9412 MWh) and SBO (500.9873 MWh), and significantly better than PSO (501.7297 MWh). Considering the average energy losses, the PO has an average energy loss of 501.2119 MWh, which is better than DE (501.2314 MWh) and SBO (501.4749 MWh), and significantly outperforms PSO (503.0422 MWh) recording improvement of 0.0039%, 0.36% and 0.0524%, respectively. The PO method achieves a maximum energy loss of 501.9493 MWh, performing better than PSO with 505.0854 MWh and SBO with 502.2436 MWh, but slightly worse than DE, which achieves 501.5581 MWh. This represents an improvement of 0.078% compared to SBO and 0.62% compared to PSO. Regarding consistency, PO has a standard deviation of 0.323993, which is lower than the 0.819117 of PSO but higher than the 0.166738 of DE and the 0.330259 of SBO. Therefore, PO outperforms PSO, DE, and SBO regarding the minimum and average energy losses, with slight improvements over DE and SBO but a substantial reduction compared to PSO.

### Second grid: IEEE 69-node

**Fig 7:**
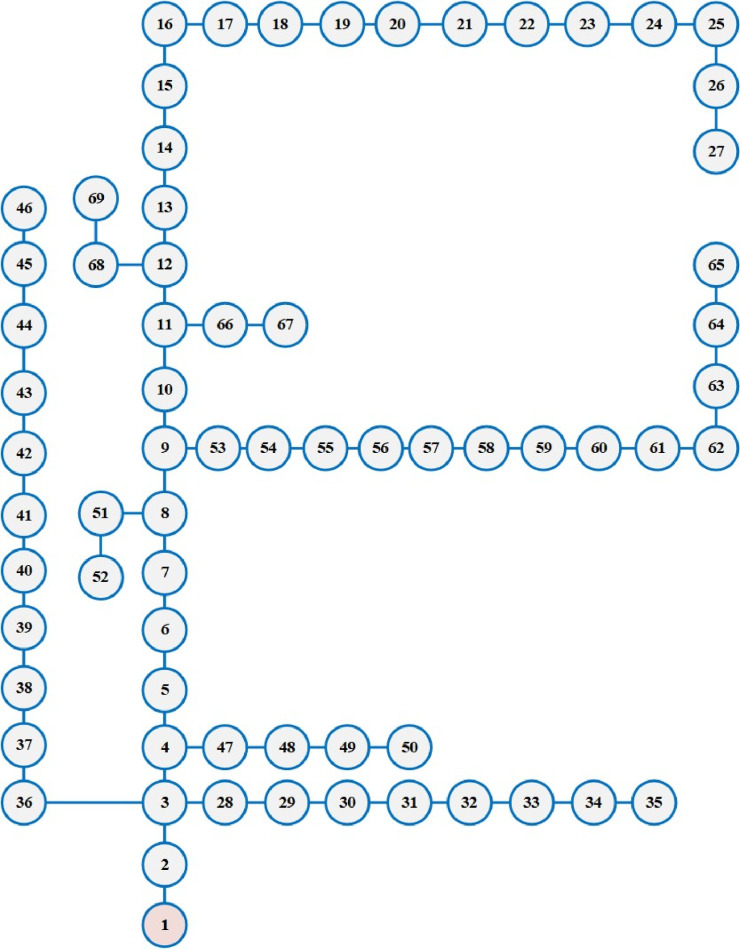
IEEE 69 node system.

Another grid under analysis comprises 68 branches and 69 buses. Fig 7 shows a single-line scheme of the test grid. It is operating at a nominal voltage of 12.66 kV [42]. Under normal load conditions, the whole active power measures 3802 kW, and the reactive power measures 2694 kVAr [43].

**Fig 8:**
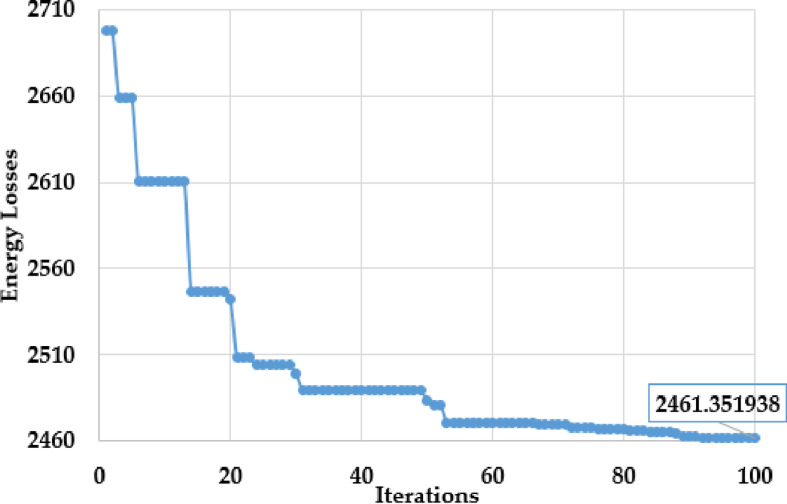
Convergences of PO algorithm for the IEEE 69 node grid.

To reduce energy loss, the suggested PO algorithm recommends connecting the solar PV systems to the second test grid. Table 4 illustrates how these units are optimally allocated, while Fig 8 exhibits the convergence fetures of the suggested PO algorithm for this scenario. The proposed PO method allocates these systems to buses 22, 64, and 61, corresponding ratings of 365 kW, 447 kW, and 1722 kW. As a result, the proposed enhanced PO method reduces the energy losses from 3784.568 MWh to 2461.352 MWh, marking a notable decrease of 34.96% in energy losses related to the initial state.

**Table 4:** Achieved outcomes allocations of solar PV units’ allocation by PO algorithm for IEEE 69 node system.

Parameters	Initial	Placement (bus)	Capacity (kW)
Solar PV units’ integration	–	64	447
	–	22	365
	–	61	1722
Total photovoltaic installed Capacity	–	2534 kW	
Energy losses (MWh)	3784.568	2461.351938	

**Fig 9:**
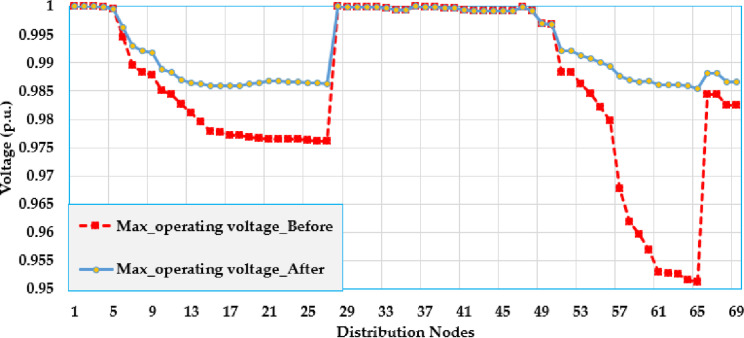
Maximum voltage values over the grid using PO algorithm of the IEEE 69 node grid.

**Fig 10:**
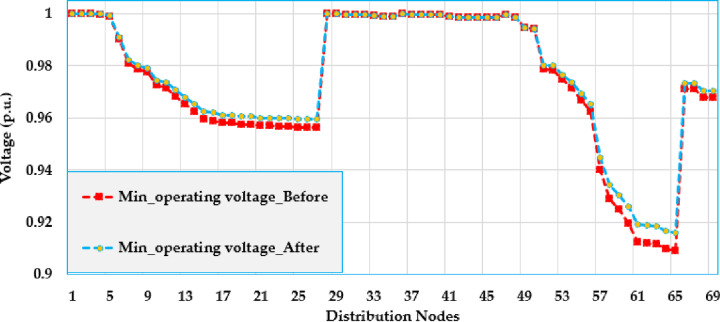
Minimum voltage values over the grid using PO algorithm of the IEEE 69 node system.

Figs 9 and 10 display the voltage change within the grid by displaying the voltage values at different nodes within the distribution grid. These figures illustrate the lowest and highest voltage magnitudes noted on each bus. The installation of solar PV systems has a noticeable effect on voltage levels, demonstrating significant improvements over the base case. Specifically, the buses 7-28 are improved to be more closely to unity. Also, the buses (52–65) at the far end from the substation are greatly raised where the voltage at node 65 is increased from 0951 PU to 0.985 PU with an improvement percentage of 3.57%.

To perform a comparative analysis of the suggested PO method, Table 5 is provided to show the energy losses attained by the PSO, SBO, DE, and PO approaches. As displayed, the PO achieves the lowest minimum energy loss at 2461.3519 MWh, outperforming all other algorithms. DE comes close with 2463.0651 MWh, and SBO is slightly higher at 2463.3289 MWh, while PSO performs significantly worse at 2477.0810 MWh. Also, the PO has an average energy loss of 2467.5107 MWh, which is better than DE (2472.8694 MWh), SBO (2478.6761 MWh), and far superior to PSO (2519.6852 MWh). The percentage reduction in minimum energy loss is 0.22% compared to DE, 2.07% compared to PSO and 0.45% compared to SBO. The PO method achieves a maximum energy loss of 2499.8619 MWh, performing better than PSO with 2550.291 MWh and SBO with 2499.8796 MWh, but slightly worse than DE, which achieves 2484.3701 MWh. In terms of maximum energy losses, PO slightly underperforms compared to DE but still significantly outperforms PSO and SBO. The standard deviation of PO is smaller than PSO and SBO, indicating better stability, though DE has the most consistent results. Overall, the PO demonstrates excellent performance in minimizing energy losses while maintaining a high level of stability, making it an efficient and reliable optimization tool for the IEEE 69-node system.

**Table 5:** Comparisons between the PO algorithm with reported techniques for the IEEE 69 node system.

Algorithms	Energy losses (MWh)				
	Minimum	Average	Maximum	Standard Deviation	Percentage Improvement based on the average losses
DE [33]	2463.065	2472.869	2484.370	5.062	0.217%
PSO [33]	2477.081	2519.685	2550.291	19.836	2.071%
SBO [33]	2463.329	2478.676	2499.880	11.678	0.450%
PO Algorithm	2461.352	2467.511	2499.862	7.820	–

## Discussion on the results

The PO uniquely addresses challenges in PV system integration by employing hyper-heuristic mechanisms inspired by pelican hunting behavior. This enables the algorithm to dynamically balance exploration and exploitation, effectively navigating complex search spaces. Unlike traditional methods, PO adapts its strategy to problem characteristics, ensuring optimal solutions even in highly constrained environments. While bio-inspired algorithms like PSO and DE have been widely used, PO offers specific advantages. Its dynamic adaptability prevents premature convergence and ensures a more thorough exploration of the solution space. Additionally, the incorporation of phase-based adjustments enhances its capability to handle multi-modal problems. The results demonstrate that PO consistently achieves lower energy losses compared to PSO and SBO, as well as competitive performance with DE. Beyond case studies, these advantages make PO a robust candidate for various real-world applications in renewable energy integration.

The simulation results highlight PO’s superior performance, particularly in minimizing energy losses. In the Ajinde 62-node grid, PO achieved a 30.81% reduction in energy losses compared to the initial scenario, outperforming PSO and SBO. This can be attributed to PO’s efficient updating mechanism, which prevents stagnation in local optima and allows for continuous improvement. In the IEEE 69-node grid, PO achieved a 34.96% reduction in energy losses, the highest among all tested algorithms. This success is likely due to the algorithm’s ability to adapt its search radius dynamically, ensuring both global exploration and local exploitation. Several other parameters are demonstrated in this study via the PO algorithm for evaluating the overall performance and feasibility of the PV system integration, beyond just energy losses as follows:

- Voltage profile improvements: The voltage at various nodes is a crucial indicator of grid stability and reliability. The results show that the PO significantly enhances the voltage profiles, maintaining them within permissible limits (0.95 to 1.05 p.u.).
- Optimal placement and sizing of PV systems: The designed PO algorithm provides insights into the most effective buses for PV placement and their corresponding capacities, which directly influence the network’s operational efficiency.
- Power flow balancing: The designed PO algorithm ensures power flow balance and maintains system stability and prevents overloads.

Moreover, Table 6 provides quantitative data on the time and memory complexity of the PO algorithm for both test systems under study. As shown, the PO algorithm requires approximately 401.02 seconds for the Nigerian 62-bus network and 414.57 seconds for the IEEE 69-node system. This slight increase in time for the IEEE system can be attributed to its higher complexity, as it involves more nodes and branches, necessitating additional computations during optimization. The memory requirements for the Nigerian 62-bus system are 3113 MB, while the IEEE 69-node system requires 3166 MB. The maximum possible array sizes are 19898 MB and 20472 MB for the Nigerian and IEEE systems, respectively. The increase in memory usage aligns with the larger search space and higher data requirements for the IEEE system. Despite this, memory consumption remains efficient and manageable, demonstrating the scalability of the PO algorithm.

**Table 6:** Time and Memory Complexity of PO algorithm for both systems under study.

Network under study	Elapsed time	Maximum possible array	Memory used by MATLAB
Nigerian distribution system (Ajinde 62-bus network)	401.02 second	19898 MB	3113 MB
IEEE standard system (69-nodes)	414.57 second	20472 MB	3166 MB

## Limitations and future recommendations

However, some limitations are remarked where the study focuses solely on minimizing energy losses, without incorporating other objectives such as cost, reliability, or environmental factors. Also, the algorithm has been tested on two specific distribution systems (Ajinde 62-node and IEEE 69-node), which may limit generalizability to other grid configurations. Although the PO shows significant improvements in performance, the computational complexity may increase for larger networks with higher node counts.

Therefore, future recommendations can be dedicated as follows:

- Multi-Objective Optimization: Extend the PO framework to address multiple objectives, including cost minimization, reliability improvement, and environmental impact reduction.
- Dynamic Constraints: Incorporate dynamic operating conditions such as variable load profiles, fluctuating irradiance, and equipment degradation over time.
- Wider Testing: Validate the PO algorithm on diverse and larger distribution networks to establish its robustness and scalability.
- Hybrid Approaches: Explore integrating PO with other optimization techniques to enhance convergence speed and solution accuracy.
- While the current study focuses primarily on energy losses, future extensions could include analyzing and assessing environmental benefits, such as reductions in carbon emissions due to renewable energy integration.

## Conclusions

In this study, we implemented the pelican optimizer (PO) method for installing solar PV systems into radial power distribution grids. The objective function is formulated with the goal of minimizing the daily energy losses of the power distribution grids. The proposed PO approach is implemented to two distribution networks: the Ajinde 62-node system, a practical Nigerian distribution grid, and the IEEE-69 node grid. Furthermore, the hourly demand fluctuations, represented as a proportion of the maximum demand situation, are considered. The findings highlighted the exceptional effectiveness of the PO method in significantly reducing energy losses, surpassing the performance of the proposed PO algorithm as well as the Particle Swarm Optimization (PSO), Differential Evolution (DE), and Satin bowerbird optimizer (SBO) algorithms. Additionally, the PO method exhibited better average metrics and success rates, leading to improved overall network performance. The outcomes of this article highlight the promising capabilities of the proposed PO algorithm as an effective solution for optimizing the integration of solar PV systems in radial power distribution grids. Utilizing the PO algorithm can enhance system efficiency and minimize the energy losses while maintaining the operational constraints. Future studies could investigate additional improvements and adaptations of the PO algorithm and explore its potential applications in other renewable energy integration challenges within power systems.

## Supporting information

S1 Fig PO Flowchart for photovoltaic integrations in distribution feeders. (PDF)

S2 Fig Hourly loading variations. (PDF)

S3 Fig Single-Line scheme of Ajinde 62-node grid. (PDF)

S4 Fig Convergences of PO algorithm for the Ajinde 62-node Nigerian grid. (PDF)

S5 Fig Maximum voltage values over the grid using PO algorithm of the Ajinde 62-bus Nigerian system. (PDF)

S6 Fig Minimum voltage values over the grid using PO algorithm of the Ajinde 62-bus Nigerian system. (PDF)

S7 Fig IEEE 69 node system. (PDF)

S8 Fig Convergences of PO algorithm for the IEEE 69 node grid. (PDF)

S9 Fig Maximum voltage values over the grid using PO algorithm of the IEEE 69 node grid. (PDF)

S10 Fig Minimum voltage values over the grid using PO algorithm of the IEEE 69 node system. (PDF)
