# Supplementary figures and images for: Performance of pelican optimizer for energy losses minimization via optimal photovoltaic systems in distribution feeders
Source: PLoS One. 2025 Mar 12;20(3):e0319298. doi: 10.1371/journal.pone.0319298 (PMC11902084; doi:10.1371/journal.pone.0319298)

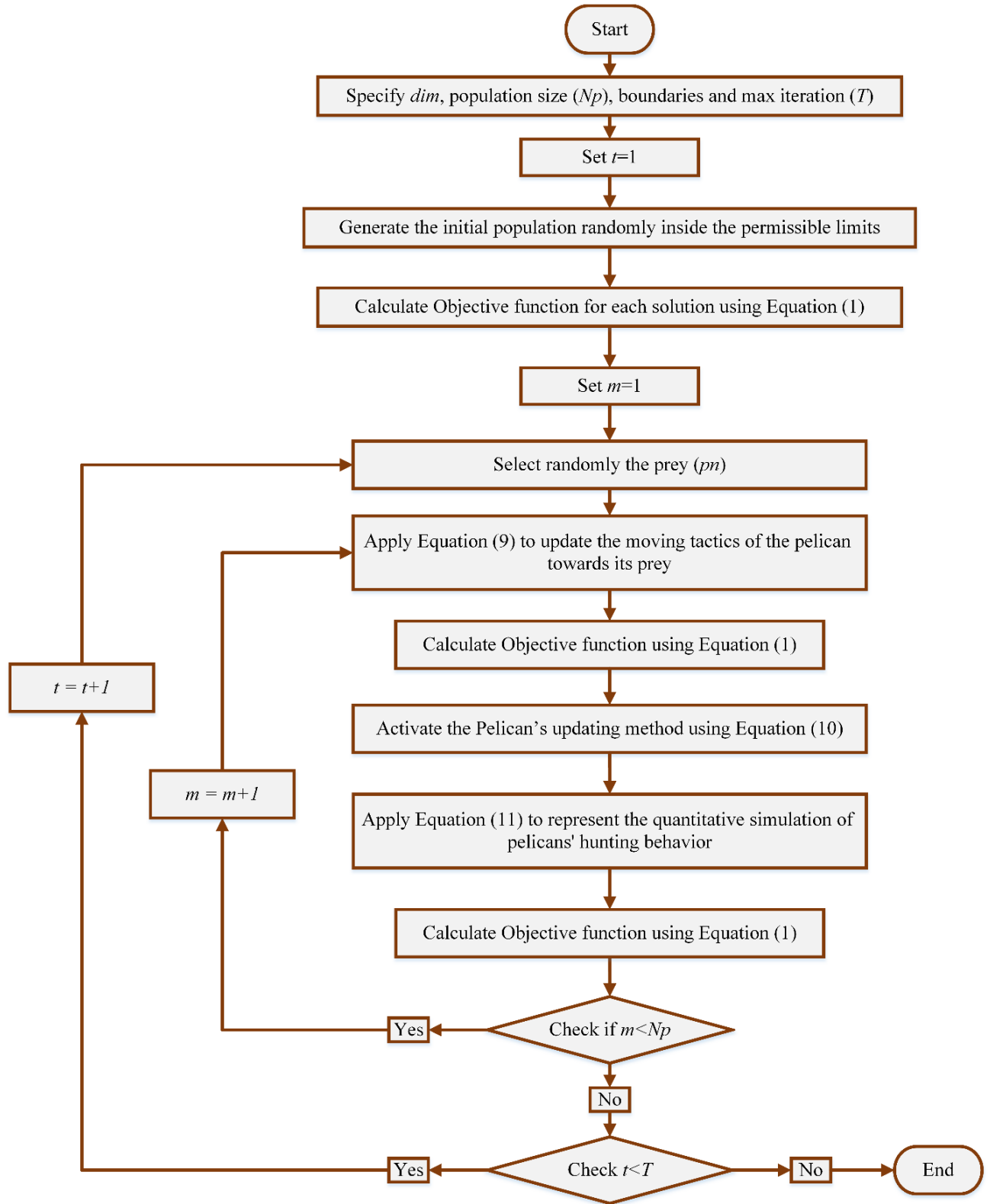

**Figure 1:** PO Flowchart for photovoltaic integrations in distribution feeders

Supplement: S1 Fig — (PDF) [file pone.0319298.s001.pdf]

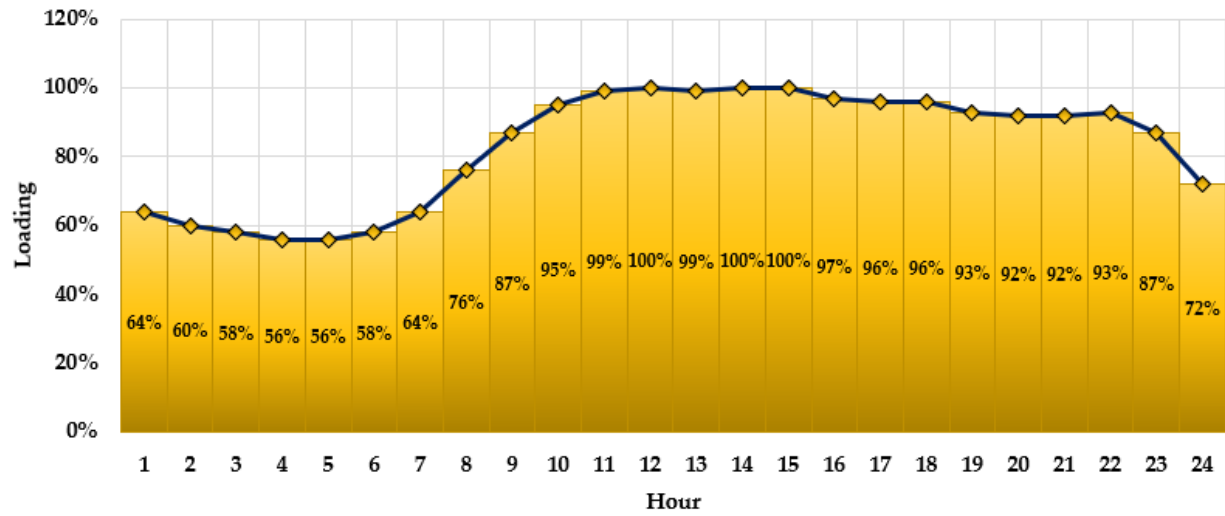

**Figure 2:** Hourly loading variations.

| Hour | Loading (%) |
|------|-------------|
| 1    | 64.0%       |
| 2    | 60.0%       |
| 3    | 58.0%       |
| 4    | 56.0%       |
| 5    | 56.0%       |
| 6    | 58.0%       |
| 7    | 64.0%       |
| 8    | 76.0%       |
| 9    | 87.0%       |
| 10   | 95.0%       |
| 11   | 99.0%       |
| 12   | 100.0%      |
| 13   | 99.0%       |
| 14   | 100.0%      |
| 15   | 100.0%      |
| 16   | 97.0%       |
| 17   | 96.0%       |
| 18   | 96.0%       |
| 19   | 93.0%       |
| 20   | 92.0%       |
| 21   | 92.0%       |
| 22   | 93.0%       |
| 23   | 87.0%       |
| 24   | 72.0%       |

Supplement: S2 Fig — (PDF) [file pone.0319298.s002.pdf]

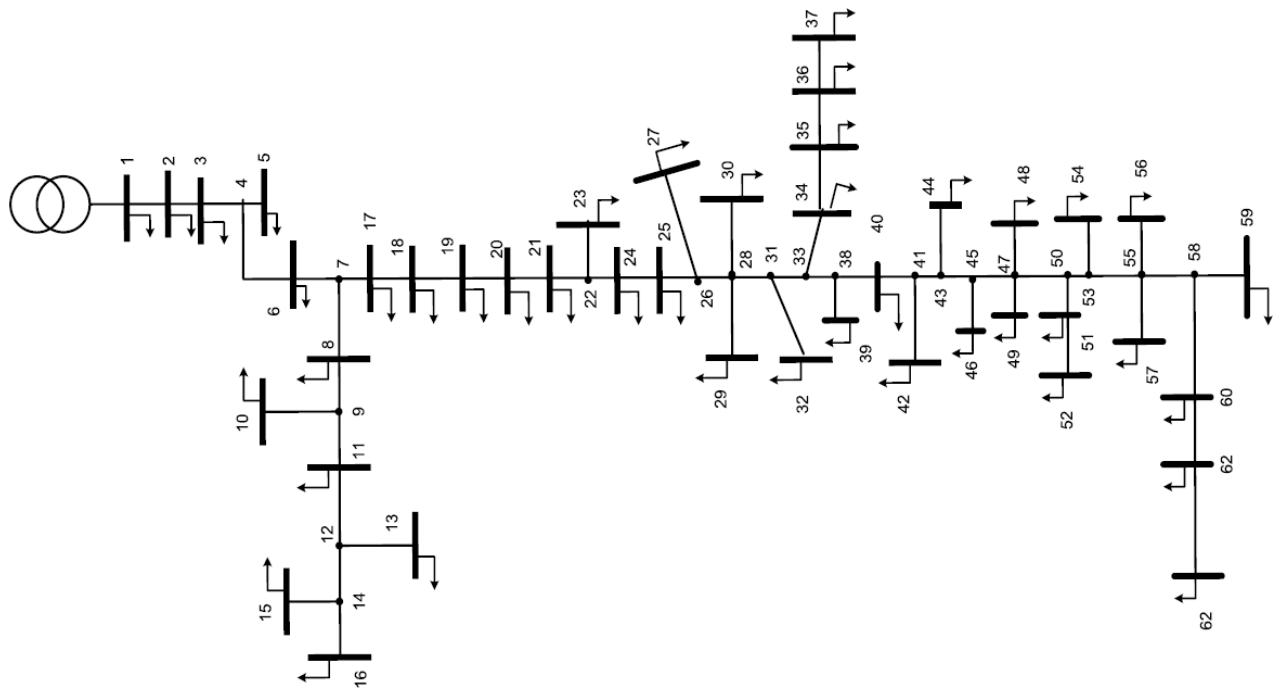

**Figure 3:** Single-Line scheme of Ajinde 62-node grid

Supplement: S3 Fig — (PDF) [file pone.0319298.s003.pdf]

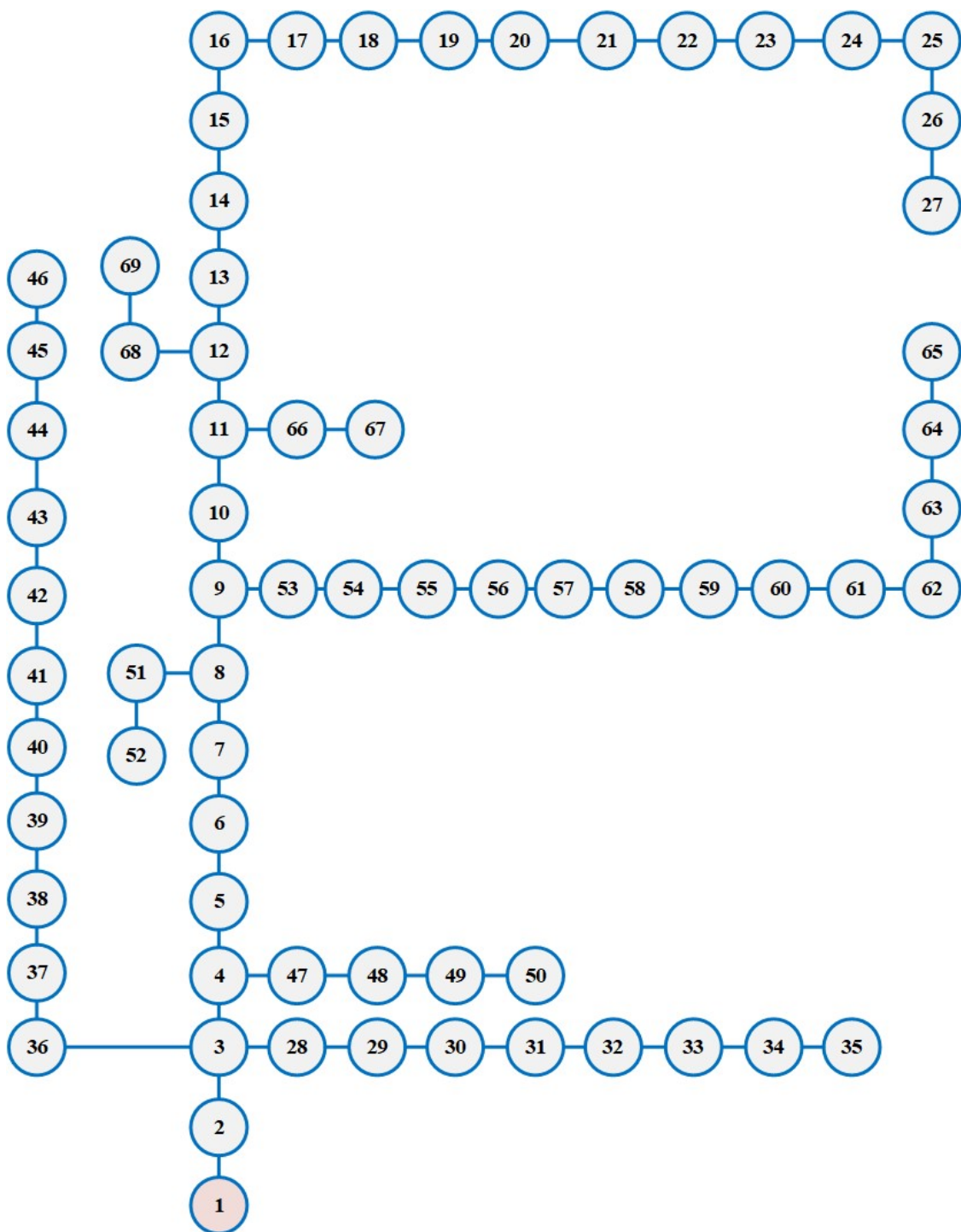

**Figure 7:** IEEE 69 node system

Supplement: S7 Fig — (PDF) [file pone.0319298.s007.pdf]

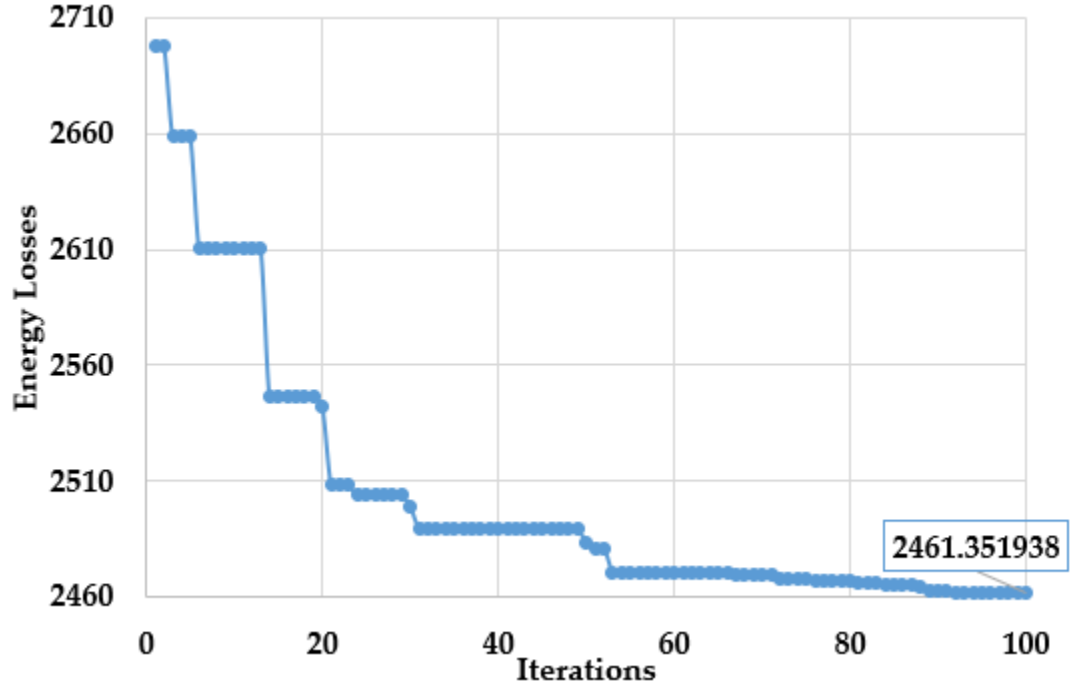

**Figure 8:** Convergences of PO algorithm for the IEEE 69 node grid

| Iterations | Energy Losses |
|------------|---------------|
| 1          | 2698.255682   |
| 2          | 2698.255682   |
| 3          | 2658.900977   |
| 4          | 2658.900977   |
| 5          | 2658.900977   |
| 6          | 2610.307601   |
| 7          | 2610.307601   |
| 8          | 2610.307601   |
| 9          | 2610.307601   |
| 10         | 2610.307601   |
| 11         | 2610.307601   |
| 12         | 2610.307601   |
| 13         | 2610.307601   |
| 14         | 2546.314575   |
| 15         | 2546.314575   |
| 16         | 2546.314575   |
| 17         | 2546.314575   |
| 18         | 2546.314575   |
| 19         | 2546.314575   |

|    |             |
|----|-------------|
| 20 | 2541.885055 |
| 21 | 2508.033183 |
| 22 | 2508.033183 |
| 23 | 2508.033183 |
| 24 | 2503.797964 |
| 25 | 2503.797964 |
| 26 | 2503.797964 |
| 27 | 2503.797964 |
| 28 | 2503.797964 |
| 29 | 2503.797964 |
| 30 | 2498.778047 |
| 31 | 2489.169219 |
| 32 | 2489.169219 |
| 33 | 2489.169219 |
| 34 | 2489.169219 |
| 35 | 2489.169219 |
| 36 | 2489.169219 |
| 37 | 2489.169219 |
| 38 | 2489.169219 |
| 39 | 2489.169219 |
| 40 | 2489.169219 |
| 41 | 2489.169219 |
| 42 | 2489.169219 |
| 43 | 2489.169219 |
| 44 | 2489.169219 |
| 45 | 2489.169219 |
| 46 | 2489.169219 |
| 47 | 2489.169219 |
| 48 | 2489.169219 |
| 49 | 2489.169219 |
| 50 | 2483.59618  |
| 51 | 2480.774021 |
| 52 | 2480.774021 |
| 53 | 2470.124283 |
| 54 | 2470.124283 |
| 55 | 2470.124283 |
| 56 | 2470.124283 |
| 57 | 2470.124283 |
| 58 | 2470.124283 |
| 59 | 2470.124283 |
| 60 | 2470.124283 |

|     |             |
|-----|-------------|
| 61  | 2470.124283 |
| 62  | 2470.124283 |
| 63  | 2470.124283 |
| 64  | 2470.124283 |
| 65  | 2470.124283 |
| 66  | 2470.124283 |
| 67  | 2469.900622 |
| 68  | 2469.900622 |
| 69  | 2469.900622 |
| 70  | 2469.900622 |
| 71  | 2469.900622 |
| 72  | 2467.663365 |
| 73  | 2467.663365 |
| 74  | 2467.663365 |
| 75  | 2467.663365 |
| 76  | 2467.191779 |
| 77  | 2466.57913  |
| 78  | 2466.57913  |
| 79  | 2466.57913  |
| 80  | 2466.57913  |
| 81  | 2465.812628 |
| 82  | 2465.812628 |
| 83  | 2465.812628 |
| 84  | 2465.175391 |
| 85  | 2465.040777 |
| 86  | 2465.040777 |
| 87  | 2464.871273 |
| 88  | 2464.083568 |
| 89  | 2462.939537 |
| 90  | 2462.939537 |
| 91  | 2462.939537 |
| 92  | 2461.560886 |
| 93  | 2461.560886 |
| 94  | 2461.560886 |
| 95  | 2461.560886 |
| 96  | 2461.560886 |
| 97  | 2461.560886 |
| 98  | 2461.559312 |
| 99  | 2461.351938 |
| 100 | 2461.351938 |

Supplement: S8 Fig — (PDF) [file pone.0319298.s008.pdf]
