# Supplementary material for: Performance of pelican optimizer for energy losses minimization via optimal photovoltaic systems in distribution feeders
Source: PLoS One. 2025 Mar 12;20(3):e0319298. doi: 10.1371/journal.pone.0319298 (PMC11902084; doi:10.1371/journal.pone.0319298)
Supplement: S4 Fig — (PDF) [file pone.0319298.s004.pdf]

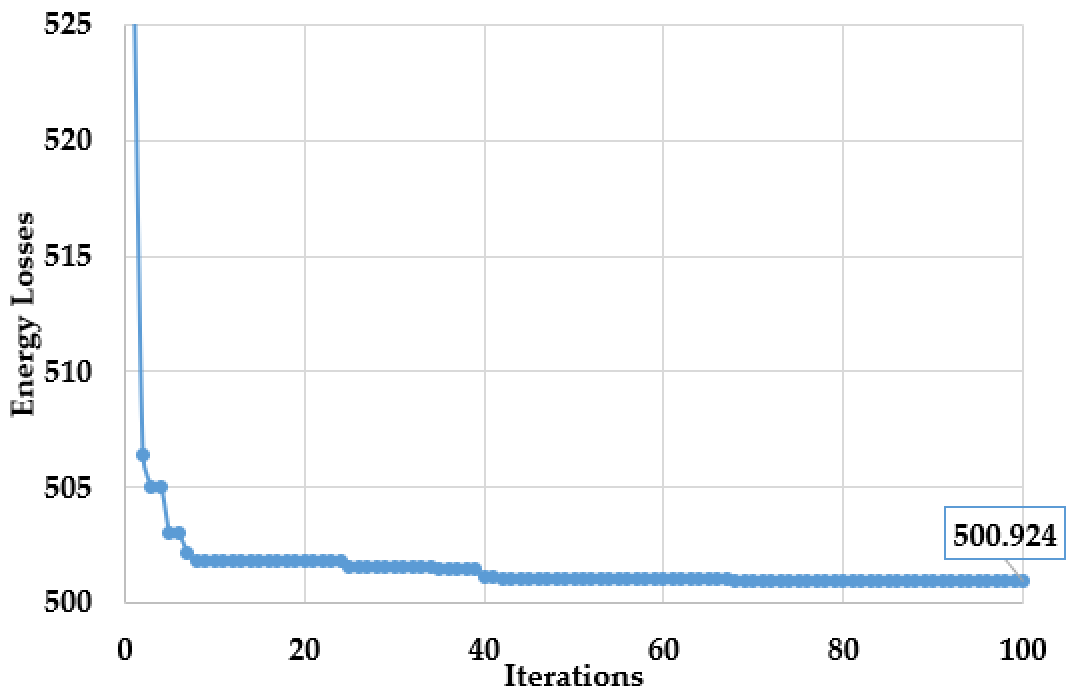

Figure 4. Convergences of PO algorithm for the Ajinde 62-node Nigerian grid

Iterations	Energy Losses
1	526.9624
2	506.371
3	505.0492
4	505.0492
5	502.9964
6	502.9964
7	502.1627
8	501.8302
9	501.8302
10	501.8302
11	501.8302
12	501.8302
13	501.8302
14	501.8292
15	501.8292
16	501.8292
17	501.8292
18	501.8292
19	501.8292

20	501.8292
21	501.7782
22	501.7782
23	501.7782
24	501.7782
25	501.5562
26	501.5545
27	501.5545
28	501.5545
29	501.5545
30	501.5396
31	501.5396
32	501.5396
33	501.5396
34	501.5396
35	501.4907
36	501.4907
37	501.4907
38	501.4665
39	501.4665
40	501.1044
41	501.1044
42	501.0172
43	501.0172
44	501.0172
45	501.0172
46	501.0172
47	501.0172
48	501.0172
49	501.0172
50	501.0172
51	501.0172
52	501.0172
53	501.0172
54	501.0172
55	501.0172
56	501.0172
57	501.0172
58	501.0172
59	501.0141
60	501.0141

61	501.0141
62	501.0141
63	501.0141
64	501.0141
65	501.0141
66	501.0141
67	501.0141
68	500.9328
69	500.9328
70	500.9328
71	500.9328
72	500.9328
73	500.9328
74	500.9328
75	500.9328
76	500.9328
77	500.9328
78	500.9328
79	500.9328
80	500.9328
81	500.9328
82	500.9312
83	500.9312
84	500.9312
85	500.9312
86	500.9312
87	500.9307
88	500.9283
89	500.9283
90	500.9283
91	500.9279
92	500.9279
93	500.9279
94	500.9279
95	500.9279
96	500.9279
97	500.9273
98	500.9273
99	500.9246
100	500.924
