# Supplementary material for: Performance of pelican optimizer for energy losses minimization via optimal photovoltaic systems in distribution feeders
Source: PLoS One. 2025 Mar 12;20(3):e0319298. doi: 10.1371/journal.pone.0319298 (PMC11902084; doi:10.1371/journal.pone.0319298)
Supplement: S5 Fig — (PDF) [file pone.0319298.s005.pdf]

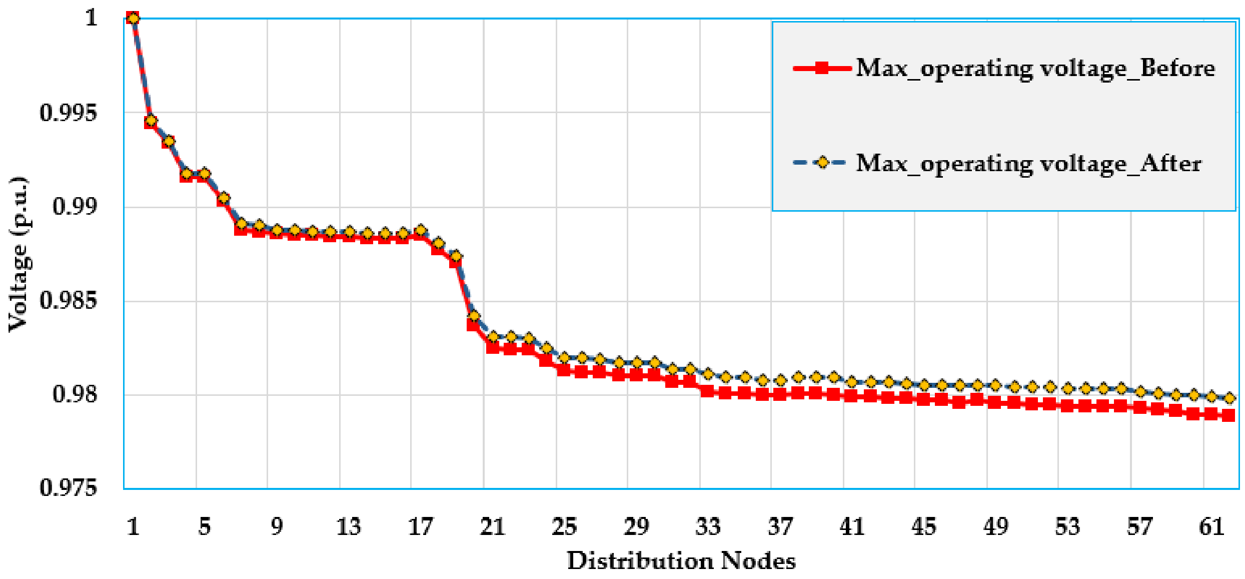

Figure 5: Maximum voltage values over the grid using PO algorithm of the Ajinde 62-bus Nigerian system

**Distribution Nodes**	Max_operating voltage_Before	Max_operating voltage_After
1	1	1
2	0.9944	0.9946
3	0.9934	0.9935
4	0.9916	0.9918
5	0.9916	0.9918
6	0.9903	0.9905
7	0.9888	0.9891
8	0.9887	0.989
9	0.9886	0.9888
10	0.9885	0.9888
11	0.9885	0.9887
12	0.9884	0.9887
13	0.9884	0.9887
14	0.9883	0.9886
15	0.9883	0.9886
16	0.9883	0.9886
17	0.9885	0.9888
18	0.9877	0.9881
19	0.987	0.9874
20	0.9837	0.9842
21	0.9825	0.9831
22	0.9824	0.9831
23	0.9824	0.983
24	0.9818	0.9825

25	0.9813	0.982
26	0.9812	0.982
27	0.9812	0.9819
28	0.981	0.9817
29	0.981	0.9817
30	0.981	0.9817
31	0.9807	0.9814
32	0.9807	0.9814
33	0.9802	0.9811
34	0.9801	0.9809
35	0.9801	0.9809
36	0.98	0.9808
37	0.98	0.9808
38	0.9801	0.9809
39	0.9801	0.9809
40	0.98	0.9809
41	0.9799	0.9807
42	0.9799	0.9807
43	0.9798	0.9807
44	0.9798	0.9806
45	0.9797	0.9805
46	0.9797	0.9805
47	0.9796	0.9805
48	0.9797	0.9805
49	0.9796	0.9805
50	0.9796	0.9804
51	0.9795	0.9804
52	0.9795	0.9804
53	0.9794	0.9803
54	0.9794	0.9803
55	0.9794	0.9803
56	0.9794	0.9803
57	0.9793	0.9802
58	0.9792	0.9801
59	0.9791	0.98
60	0.979	0.98
61	0.979	0.9799
62	0.9789	0.9798
