# Supplementary material for: Performance of pelican optimizer for energy losses minimization via optimal photovoltaic systems in distribution feeders
Source: PLoS One. 2025 Mar 12;20(3):e0319298. doi: 10.1371/journal.pone.0319298 (PMC11902084; doi:10.1371/journal.pone.0319298)
Supplement: S6 Fig — (PDF) [file pone.0319298.s006.pdf]

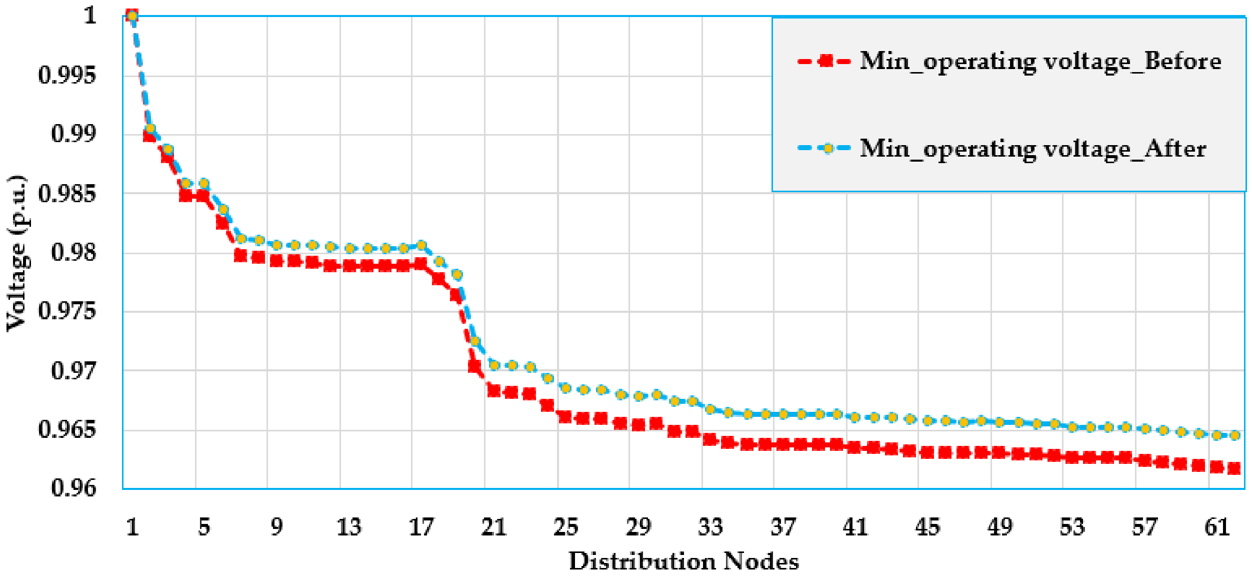

Figure 6: Minimum voltage values over the grid using PO algorithm of the Ajinde 62-bus Nigerian system

**Distribution Nodes**	Min_operating voltage_Before	Min_operating voltage_After
1	1	1
2	0.9899	0.9906
3	0.988	0.9888
4	0.9848	0.9859
5	0.9848	0.9859
6	0.9824	0.9837
7	0.9797	0.9812
8	0.9796	0.981
9	0.9792	0.9807
10	0.9792	0.9807
11	0.9791	0.9806
12	0.9789	0.9805
13	0.9789	0.9804
14	0.9788	0.9804
15	0.9788	0.9803
16	0.9788	0.9803
17	0.979	0.9806
18	0.9777	0.9793
19	0.9764	0.9781
20	0.9703	0.9725
21	0.9682	0.9705
22	0.9681	0.9704
23	0.968	0.9703
24	0.967	0.9694
25	0.9661	0.9685

26	0.9659	0.9684
27	0.9659	0.9684
28	0.9655	0.968
29	0.9654	0.9679
30	0.9655	0.968
31	0.9649	0.9674
32	0.9649	0.9674
33	0.9641	0.9667
34	0.9639	0.9665
35	0.9638	0.9664
36	0.9637	0.9663
37	0.9637	0.9663
38	0.9638	0.9664
39	0.9638	0.9664
40	0.9637	0.9664
41	0.9635	0.9661
42	0.9634	0.9661
43	0.9633	0.966
44	0.9632	0.9659
45	0.9631	0.9658
46	0.9631	0.9658
47	0.963	0.9657
48	0.9631	0.9658
49	0.963	0.9657
50	0.9629	0.9656
51	0.9629	0.9655
52	0.9628	0.9655
53	0.9626	0.9653
54	0.9626	0.9653
55	0.9626	0.9653
56	0.9626	0.9653
57	0.9623	0.9651
58	0.9622	0.965
59	0.9621	0.9648
60	0.9619	0.9647
61	0.9618	0.9645
62	0.9617	0.9645
