# Supplementary material for: Performance of pelican optimizer for energy losses minimization via optimal photovoltaic systems in distribution feeders
Source: PLoS One. 2025 Mar 12;20(3):e0319298. doi: 10.1371/journal.pone.0319298 (PMC11902084; doi:10.1371/journal.pone.0319298)
Supplement: S9 Fig — (PDF) [file pone.0319298.s009.pdf]

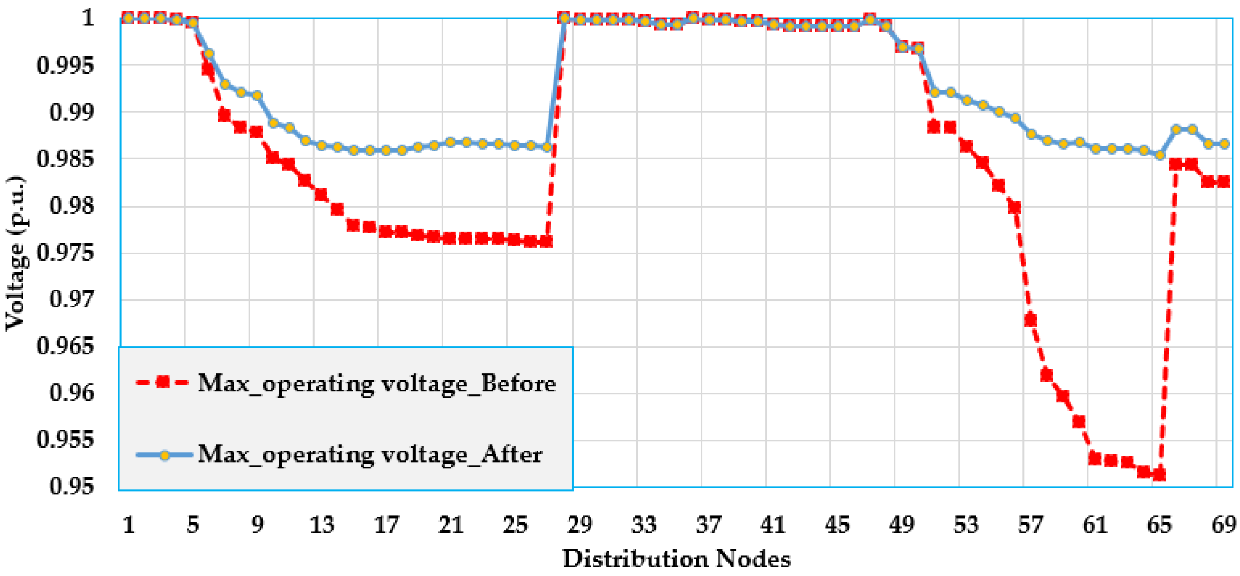

Figure 9. Maximum voltage values over the grid using PO algorithm of the IEEE 69 node grid

**Distribution Nodes**	Max_operating voltage_Before	Max_operating voltage_After
1	1	1
2	1	1
3	1	1
4	0.9999	0.9999
5	0.9995	0.9995
6	0.9946	0.9963
7	0.9896	0.9929
8	0.9884	0.9922
9	0.9878	0.9918
10	0.985	0.9889
11	0.9844	0.9883
12	0.9827	0.9869
13	0.9811	0.9865
14	0.9795	0.9862
15	0.9779	0.9859
16	0.9777	0.9859
17	0.9772	0.9859
18	0.9772	0.9859
19	0.9769	0.9863
20	0.9767	0.9865
21	0.9765	0.9868
22	0.9765	0.9868
23	0.9764	0.9867
24	0.9764	0.9866

25	0.9763	0.9864
26	0.9762	0.9864
27	0.9762	0.9863
28	1	1
29	0.9999	0.9999
30	0.9999	0.9999
31	0.9998	0.9998
32	0.9998	0.9998
33	0.9996	0.9996
34	0.9994	0.9994
35	0.9994	0.9994
36	1	1
37	0.9999	0.9999
38	0.9998	0.9998
39	0.9997	0.9997
40	0.9997	0.9997
41	0.9994	0.9994
42	0.9992	0.9992
43	0.9992	0.9992
44	0.9992	0.9992
45	0.9991	0.9991
46	0.9991	0.9991
47	0.9999	0.9999
48	0.9992	0.9992
49	0.997	0.997
50	0.9967	0.9967
51	0.9884	0.9921
52	0.9884	0.9921
53	0.9863	0.9913
54	0.9846	0.9907
55	0.9822	0.99
56	0.9798	0.9893
57	0.9678	0.9877
58	0.9619	0.987
59	0.9596	0.9867
60	0.9569	0.9868
61	0.9529	0.9861
62	0.9528	0.9861
63	0.9526	0.9861
64	0.9515	0.986
65	0.9512	0.9854

66	0.9844	0.9882
67	0.9844	0.9882
68	0.9825	0.9866
69	0.9825	0.9866
