# Supplementary material for: Performance of pelican optimizer for energy losses minimization via optimal photovoltaic systems in distribution feeders
Source: PLoS One. 2025 Mar 12;20(3):e0319298. doi: 10.1371/journal.pone.0319298 (PMC11902084; doi:10.1371/journal.pone.0319298)
Supplement: S10 Fig — (PDF) [file pone.0319298.s010.pdf]

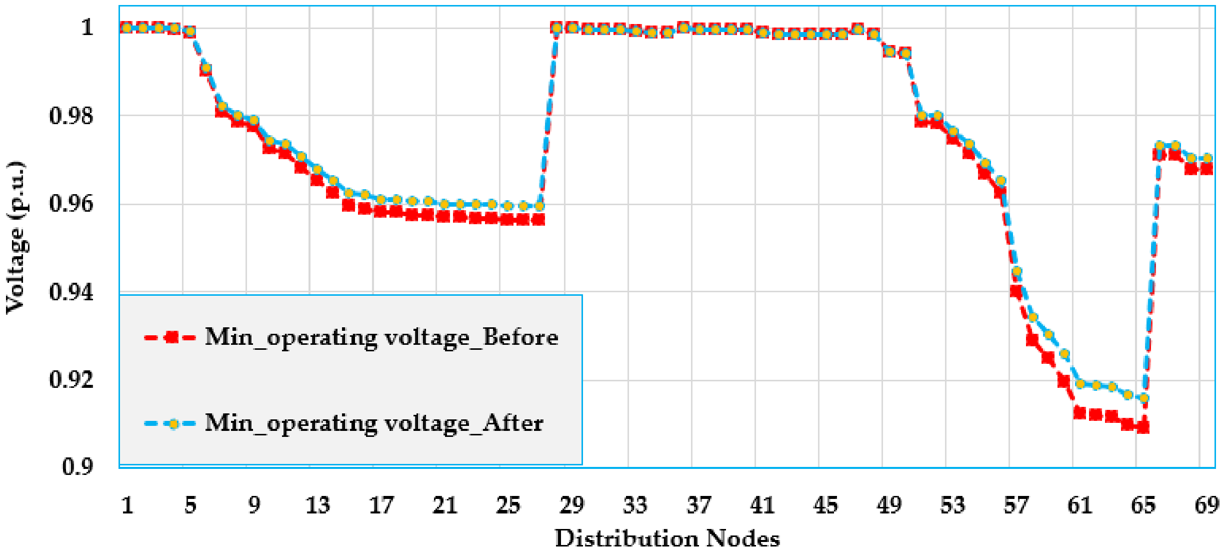

Figure 10. Minimum voltage values over the grid using PO algorithm of the IEEE 69 node system

**Distribution Nodes**	Min_operating voltage_Before	Min_operating voltage_After
1	1	1
2	1	1
3	0.9999	0.9999
4	0.9998	0.9999
5	0.999	0.9991
6	0.9901	0.9908
7	0.9808	0.9822
8	0.9786	0.9802
9	0.9775	0.9791
10	0.9725	0.9745
11	0.9714	0.9735
12	0.9682	0.9706
13	0.9653	0.9679
14	0.9624	0.9652
15	0.9595	0.9625
16	0.959	0.962
17	0.9581	0.9612
18	0.9581	0.9612
19	0.9576	0.9608
20	0.9573	0.9605
21	0.9569	0.9601
22	0.9569	0.9601
23	0.9568	0.96
24	0.9566	0.9598
25	0.9565	0.9597

26	0.9564	0.9596
27	0.9564	0.9596
28	0.9999	0.9999
29	0.9999	0.9999
30	0.9997	0.9997
31	0.9997	0.9997
32	0.9996	0.9996
33	0.9993	0.9994
34	0.999	0.999
35	0.9989	0.999
36	0.9999	0.9999
37	0.9997	0.9998
38	0.9996	0.9996
39	0.9995	0.9996
40	0.9995	0.9996
41	0.9988	0.9989
42	0.9986	0.9986
43	0.9985	0.9985
44	0.9985	0.9985
45	0.9984	0.9984
46	0.9984	0.9984
47	0.9998	0.9998
48	0.9985	0.9986
49	0.9947	0.9947
50	0.9942	0.9942
51	0.9786	0.9802
52	0.9785	0.9802
53	0.9747	0.9766
54	0.9714	0.9736
55	0.9669	0.9695
56	0.9626	0.9654
57	0.9401	0.9447
58	0.929	0.9345
59	0.9248	0.9305
60	0.9197	0.9259
61	0.9123	0.9191
62	0.9121	0.9188
63	0.9117	0.9184
64	0.9098	0.9167
65	0.9092	0.9161
66	0.9713	0.9734

67	0.9713	0.9734
68	0.9679	0.9703
69	0.9679	0.9703
